# Building a genetic risk model for bipolar disorder from genome-wide association data with random forest algorithm

**DOI:** 10.1038/srep39943

**Published:** 2017-01-03

**Authors:** Li-Chung Chuang, Po-Hsiu Kuo

**Affiliations:** 1 Department of Nursing, Cardinal Tien Junior College of Healthcare & Management, I-Lan, Taiwan; 2 Department of Public Health & Institute of Epidemiology and Preventive Medicine, College of Public Health, National Taiwan University, Taipei, Taiwan; 3 Research Center for Genes, Environment and Human Health, National Taiwan University, Taipei, Taiwan

## Abstract

A genetic risk score could be beneficial in assisting clinical diagnosis for complex diseases with high heritability. With large-scale genome-wide association (GWA) data, the current study constructed a genetic risk model with a machine learning approach for bipolar disorder (BPD). The GWA dataset of BPD from the Genetic Association Information Network was used as the training data for model construction, and the Systematic Treatment Enhancement Program (STEP) GWA data were used as the validation dataset. A random forest algorithm was applied for pre-filtered markers, and variable importance indices were assessed. 289 candidate markers were selected by random forest procedures with good discriminability; the area under the receiver operating characteristic curve was 0.944 (0.935–0.953) in the training set and 0.702 (0.681–0.723) in the STEP dataset. Using a score with the cutoff of 184, the sensitivity and specificity for BPD was 0.777 and 0.854, respectively. Pathway analyses revealed important biological pathways for identified genes. In conclusion, the present study identified informative genetic markers to differentiate BPD from healthy controls with acceptable discriminability in the validation dataset. In the future, diagnosis classification can be further improved by assessing more comprehensive clinical risk factors and jointly analysing them with genetic data in large samples.

An accurate risk score model has substantial benefits in assisting the early screening of diseases, patient management, and clinical diagnosis. In addition, a risk score model has been applied to the prognosis prediction for complex traits, such as cardiovascular diseases^1^,^2^ and cancers^3^. With the advances in generating and the accumulation of genetic information, especially the increasing popularity in genome-wide association (GWA) studies to provide more comprehensive information about genetic variation for the trait of interest, some studies have incorporated such data to construct risk score models for disease diagnosis, such as schizophrenia and post-traumatic stress disorder^4^,^5^,^6^,^7^.

Although many GWA studies have been conducted for heritable traits, only a few susceptible loci are reproducibly reported, with a small effect size between 1.2 and 2.0^8^,^9^. In a recent massive GWA study of schizophrenia, 108 loci were identified. However, each of these loci contributed a tiny fraction of the risk in the population; even the polygenic risk score explained less than 3.4% of the variance^10^. The general consensus is that multiple genes may act synergistically and interactively to influence the risk for complex diseases^11^. Jointly considering the main and interaction effects among multi-loci in whole-genome analyses is advantageous in constructing the genetic risk score model. Traditional statistical approaches in GWA studies usually focus on detecting the main effects without considering the non-linear patterns among markers and their interactions. Machine learning methods have fewer assumptions about analysis models, and have been frequently used in the analysis of high-dimensional data^12^. The random forest (RF) method is one commonly used algorithm in machine learning techniques. Because the hierarchical decision tree structure in RF can model non-linear associations, this method is often used to capture interactions among loci, and is likely to perform well in uncovering the genetic causes underlying the etiology of complex disorders.

The RF is an ensemble-based machine learning method that uses multiple classification and regression trees as classifiers. After taking the majority vote over all classifiers, the RF method combines information across all trees to reveal variable importance. It can assess associations and interactions simultaneously^13^. The RF has been applied to many biological studies, such as gene expression^14^, metabolomics^15^, proteomics^16^, and GWA^17^. These studies showed that the RF method provides good accuracy, less internal examination of error, and high variable importance from mass biological data. Thus, important variables (e.g. biomarkers or SNPs in genetic data) that are selected by the RF procedure form a useful basis to construct risk score models for the risk of developing complex disorders.

In the current study, we focused on a complex psychiatric trait, bipolar disorder (BPD) with a high heritability of around 0.6 to 0.8^18^,^19^, as the target for the risk score model construction. BPD is a severe psychiatric disorder, which affects approximately one in a hundred people worldwide. Many studies have suggested that the prevalence of BPD has increased worldwide in the last decade^20^,^21^,^22^. Without proper treatment, 15% of patients committed suicide^23^. Patients with BPD experience intermittent manic and depressive episodes, and usually exhibit chronic courses. Several large-scale GWA studies have been conducted to provide a list of susceptible genetic loci for BPD, without considering the join or interaction effects among loci^8^,^24^,^25^. So far, reliable genetic markers or objective biological indices are unavailable for clinical use in assisting diagnosis or prognosis. The diagnosis of BPD largely depends on a subjective report of patient’s syndromes and clinical characteristics.

In the present study, we used the RF-based method to construct a genetic risk prediction model for BPD, using pre-screened potentially associated markers in large-scale GWA datasets. The procedures are as follows. First, the RF method was used to select important variables as candidate risk markers in a BPD GWA dataset from the Genetic Association Information Network (GAIN). Second, the multivariable logistic regression with variable-selection methods was used to further select a smaller optimal subset of the variables from all candidate risk markers. We could then build risk score models using the identified optimal markers for the GAIN-BPD dataset. Third, to estimate the performance of the risk score models, leave-one-out cross-validation was performed for internal validation. We also used the other BPD-GWA dataset, Systematic Treatment Enhancement Program (STEP), as an external validation data. Finally, we performed a gene sets analysis to uncover the underlying biological mechanisms for those loci that were identified as the candidate risk markers.

## Result

**Figure 1:**
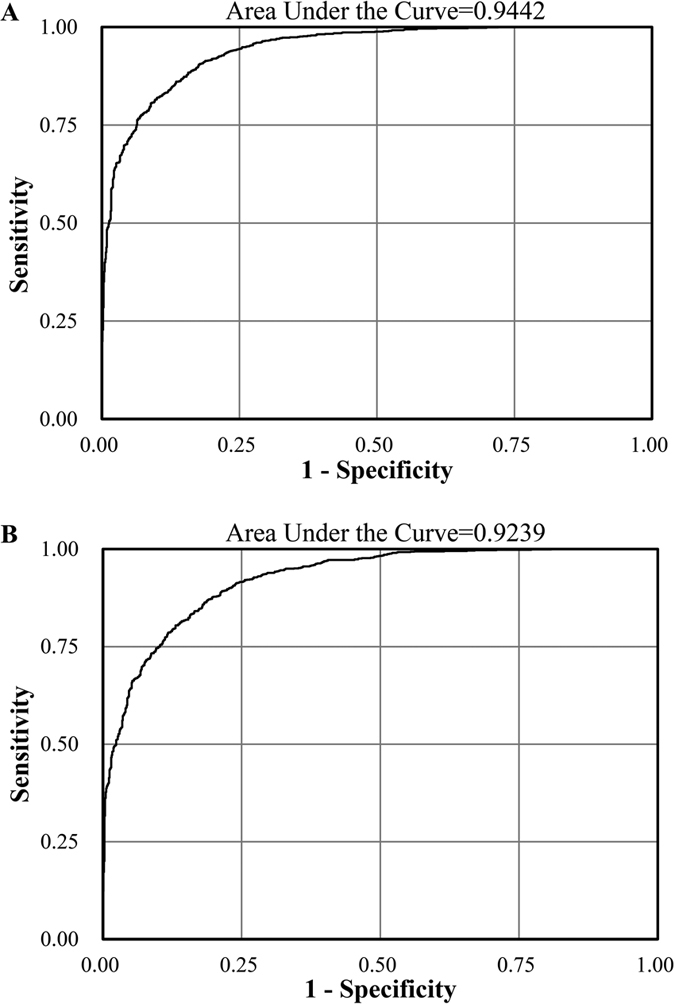
The receiver characteristic curves for (**A**) the 289 candidate markers and (**B**) the 121 optimal markers in the GAIN bipolar disorder dataset.

The accuracy of the RF procedure was evaluated in the GAIN training set created during the forest growing process. The accuracy of the RF classification in the GAIN data was 0.939 in controls and 0.852 in BPD patients. We ranked markers based on values of the two indices, the Gini Index (GI) and the conditional variable importance (VI) from the RF procedure. Because the two indices did not completely agree with each other, we used the union markers of the top ranked 200 in each index. As Table 1 shows, we included 348 single nucleotide polymorphisms (SNPs) to calculate the performance of discrimination ability. After excluding SNPs with complete linkage disequilibrium (LD), 289 SNPs were retained as the candidate risk markers in the regression model. As shown in Fig. 1A, the 289 candidate markers had a good discrimination ability with an area under the receiver operating characteristic (AUROC) of 0.944 (95% confidence interval (CI), 0.935–0.953), and calibration ability measured by the Hosmer-Lemeshow test (p-value = 0.933). The multivariable logistic regression with stepwise selection retained 121 SNPs as the final optimal marker set. A good discrimination ability was observed with an AUROC of 0.924 (95% CI, 0.913–0.935) based on the 121 markers (Fig. 1B).

The genetic risk score based on this final prediction model was calculated for each individual from accumulating numbers of the risk alleles and weighted by the beta regression coefficient. The risk scores among all participants were ranged from 143.8 to 228.4 in the GAIN dataset, with a mean of 175.4 in the controls and 191.3 in the BPD patients. To find an optimal cutoff point, we used the Youden Index to obtain the risk score cutoff as 184, and the corresponding sensitivity and specificity for BPD were 0.777 and 0.854, respectively (Table 2). The likelihood ratios of the risk score with the optimal cutoff point were 5.322 for a positive result and 0.261 for a negative result, indicating moderate evidence for the differentiation between BPD patients and healthy controls.

We then used leave-one-out cross-validation to conduct internal validation for classification accuracy (Table 3). The predicted error rates were around 0.2 using either the 289 candidate risk markers, the 121 optimal markers and the risk score. The STEP dataset was used as an external validation dataset to evaluate the performance of discrimination ability. In general, the discrimination performance was acceptable using 289 candidate risk predictors in the STEP data, which had an AUROC of 0.702 (95% CI, 0.681–0.723) and a good calibration ability (Hosmer-Lemeshow test, p-value = 0.681). With only the 121 optimal predictors, the AUROC dropped to 0.639 (95% CI, 0.617–0.662), with a low calibration ability (Hosmer-Lemeshow test, p-value = 0.0002). The risk score method showed the poorest discrimination ability (AUROC = 0.506; 95% CI, 0.482–0.529).

We performed the same set of analyses using STEP data as the training dataset, and the GAIN data as the validation. The results are displayed in Supplementary Table1. In total, 354 candidate risk markers were identified for the STEP dataset. After excluding SNPs with complete LD, 312 SNPs were retained in the regression model and had the discrimination ability with AUROC of 0.934 (95% CI, 0.925–0.944). In the external validation GAIN dataset, decreased but acceptable discrimination performance was again observed, with an AUROC of 0.732 (95% CI, 0.711–0.754) (Supplementary Table1). It is worth noting that there were no overlapping candidate risk markers between the two datasets. If we mapped all candidate risk markers from the two datasets to genes, there were in total 233 gene regions, including 98 genes in the GAIN dataset and 144 genes in the STEP dataset. Only 9 genes (3.8%) overlapped between the two datasets, including genes *ALK, TACR1, LRP1B, GALNT17, NAV2, ODZ4, RAD51L1, KTN1, and CACNG2*.

Significantly enriched gene-sets were identified for these mapped genes in the two GWA datasets of BPD. Table 4 showed that 43 pathways were identified in the GAIN dataset with a q-value of less than 0.01 after correction for multiple comparisons. In the STEP dataset, 28 significant pathways were identified. Important biological pathways were reported, including cation ion channel activity (such as voltage-gated calcium channel activity and complex, regulation of action potential and cation transport), membrane structure (such as plasma membrane, transmembrane receptor activity and establishment of location), neuron function (such as brain development, axon guidance and GABA receptor activity) and cytoskeleton (such as cytoskeletal protein binding and actin filament).

## Discussion

It is a common interest to explore the usage of genetic findings for heritable complex traits. There is an absence in the literature of a risk score model based on genetic information for the diagnosis of BPD. Non-replication across datasets is often observed, especially when focusing on specifically significant markers. Hence, using extremely significant markers in one sample to construct a genetic risk model to apply to other samples might not produce good prediction accuracy. On the other hand, informative genetic markers, which are selected by methods of machine learning, have been used for the classification of outcomes or for predicting the risk of developing diseases, such as early detection of prostate cancer^26^, treatment response in attention deficit hyperactivity disorder^27^, and identification of idiopathic autism spectrum disorder (ASD) patients^28^. Among the many machine learning methods, such as support vector machine, linear discriminant analysis, and *k*-nearest neighbour classification, RF is often applied in biomedical research with different data sources, such as gene expression^29^. Similar applications are reported using GWA datasets for complex traits with low prediction errors, such as severe asthma^30^,^31^. To our best knowledge, the present study reports the first prediction results for BPD using an RF approach to select informative markers which jointly consider the main and interaction effects among genetic variants. Our results revealed that these informative markers possess fair to good discriminability for BPD patients in the training and validation datasets.

Diagnoses of psychiatric disorders often largely depend on clinical interview rather than biomarkers. With the RF procedures, the 289 candidate risk predictors in the GAIN dataset perform well with an AUROC of 0.944 and a 0.702 AUROC in the validation dataset. Moreover, high sensitivity and specificity are also observed using the more parsimonious 121 optimal markers and the risk score, with acceptable predicted error rates less than 0.2 in leave-one-out cross-validation. Without the RF procedures, if we selected the same 289 candidate predictors based on p-values significance in the GAIN data to estimate the discriminability in the STEP dataset, the AUROC slightly dropped to 0.686 (data not shown). In the literature, some risk score models were built using genetic information in aid of improving disease classification for complex traits, without satisfactory prediction power. For instance, Golan and colleagues (2014) used the random effect approach and reported the discrimination ability with an AUROC of 0.62 for BPD patients from the Wellcome Trust Case Control Consortium dataset^32^. Using SNPs within 13 significant pathways in a study of ASD, one recent study included 237 SNPs to generate a genetic diagnostic classifier and reported an 85.6% prediction accuracy in the Central European cohort, but the accuracy dropped to 50.6% in the Han Chinese cohort^33^. Moreover, based on differentially expressed 762 unique genes, a previous study reported an 82.5% prediction accuracy for ASD^34^. In our study, the RF approach demonstrated a fine performance in selecting the informative genetic markers from massive GWA data. The classification accuracy for BPD in the current study is at the higher end with low error rates. In particular, we still obtained fair results with an AUROC of 0.702 in the STEP validation dataset.

It is commonly observed that the accuracy of the genetic prediction model is reduced in external validation samples^33^,^34^. Schulze and colleagues (2014) constructed a polygenic model for BPD, however, the performance of this model is poor in two external validation datasets, with AUROC ranged between 0.55 to 0.57^35^. The heterogeneity inherited in different studies and samples is often noted, which might reflect differences in sample ascertainment, population stratification, or experimental variations. An example of this is demonstrated in a large-scale study of Psychiatric Genomics Consortium (PGC) for population stratification. Using the multivariate linear mixed model approach, Maier and colleagues (2015) created genomic risk scores for severe psychiatric disorders, including schizophrenia, BPD, and major depressive disorder, using GWA data in PGC as the training set. In the validation data, the correlation coefficients between the observed status of the psychiatric disorders and their predicted genomic risk scores were low, ranged from 0.076–0.224^7^. To evaluate the population stratification, they calculated ancestry principle components of PGC data and then divided GWA data into four groups of the first ancestry principal component that reflect the population difference between individuals. Their results indicated significant heterogeneity for BPD in PGC GWA datasets (p-value = 0.0017), which is likely attributed to the ancestral population differences^7^. Therefore, heterogeneity derived from many sources might result in lowered prediction accuracy in an external dataset and hinders clinical usage and further application to assist diagnosis.

We ran both GWA datasets as the training and the other as the validation dataset for model construction. In either scenario, a very similar classification performance is observed, suggesting the stability of current procedures. However, we also noticed that there were no overlapping markers selected by RF in the two datasets as candidate risk markers. The agreement increased in gene levels across both datasets, where the same 9 genes are mapped in the two datasets. It may be intuitive, as genetic markers identified are often not causal variants, but rather the proxy for real causal variants. Therefore, the agreement may be the least in marker level, and the increase in gene and pathway levels, especially when heterogeneity exists among datasets. The 9 genes contained both sets of the candidate risk markers, and many studies have indicated that some of these genes are associated with BPD or brain function, such as *ODZ4*^36^, *TACR1*^37^, *KTN1*^38^ and *CACNG2*^39^. A previous GWA study using 11,974 BPD cases and 51,792 controls, identified a new intronic variant in *ODZ4*^36^. A recent study indicated that the genetic variants showed specific volumetric effects on the putamen and altered the expression of the *KTN1* gene in both brain and blood tissue^38^. Similarly, we found a number of significant pathways for the identified genes. These pathway results are quite consistent with pathways findings in previous GWA studies for bipolar disorder^40^,^41^.

There were some limitations in the present study. Although risk score models have been used to capture genetic effects from large-scale GWA studies, the power of discrimination was, however, inadequate in previous studies. Dudbridge and colleagues (2013) indicated that the power of polygenic score might be sufficient to use about 2,000 cases and controls, respectively^42^. Even with a good discrimination ability, a smaller sample size of the present study might cause the low power of classification model for BPD patients. In addition, we only used the genetic information to create the risk score model for BPD. The complex psychiatric disorders were caused by the interactions of genetic and environmental risk factors, such as substance dependence and childhood maltreatment. Wong and colleagues (2012) used a risk-classification tree analysis to create a reliable framework based on interactions of genetic variants and environmental factors^43^. Lacking the information from environmental factors might hinder the application of a prediction model for clinical diagnosis.

In conclusion, we successfully used a machine learning approach to extract informative genetic markers for the construction of a risk score model. Our results indicated a fair discrimination ability for BPD patients with AUROCs of around 0.70 in the external validation datasets. Integration of more comprehensive risk factors from family and environmental data in larger samples is necessary to construct a more precise and applicable risk score model for BPD, to assist with clinical diagnosis in the future.

## Materials and Methods

**Figure 2:**
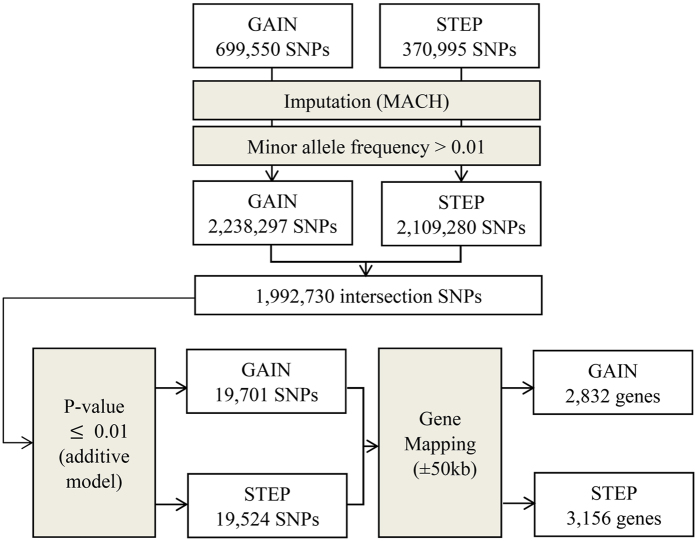
The summary description of the selection of candidate markers for the construction of the genetic risk score model. **GAIN**: the Genetic Association Information Network; **STEP**: the Systematic Treatment Enhancement Program; **MACH**: The program Markov Chain Haplotyping.

The study design and analysis flow chart are displayed in Fig. 2. Details of the datasets and analytic procedures for the selection of candidate markers in model construction are described below.

### Imputation and quality control in the GWA datasets

We used two individual GWA datasets of BPD in the Caucasian populations, the GAIN (https://dbgap.ncbi.nlm.nih.gov/aa/wga.cgi?login=&page=login)^24^ and the STEP data (https://www.nimhgenetics.org/available_data/bipolar_disorder/)^25^. The details of participant enrolment and genotyping of the two GWA studies were provided in their original articles^24^,^25^. In brief, in the GAIN dataset, individuals were Americans with European ancestry, including 1,001 BPD cases and 1,034 controls. In the STEP dataset, there were 955 BPD cases and 1,498 healthy subjects from the National Institute of Mental Health Genetics Initiative. After quality control for genotypic data, a total of 699,550 and 370,995 autosomal SNPs were retained in the GAIN and STEP datasets, respectively.

Because the two GWA studies used different genotyping platforms, we imputed all the autosomal SNPs for the two GWA datasets based on those genotyped SNPs that passed quality control, to obtain the maximal number of common SNPs for the construction of prediction models for the two datasets. We applied Markov Chain Haplotyping (MACH) 1.0^44^ to perform genotype imputation, and the HapMap II CEU (release 22) samples were used as the reference panel (www.hapmap.org). Well-imputed SNPs had squared correlations ≥0.30 between imputed and true genotypes, which were suggested by MACH. After removing markers with minor allele frequency (MAF) less than 1%, the number of well-imputed SNPs was 2,238,297 and 2,109,280 for the GAIN and the STEP datasets, respectively. In total, there were 1,992,730 well-imputed SNPs overlapped in the two datasets.

### Criteria for candidate markers in the construction of prediction models

To construct the genetic prediction model for BPD, we first performed association analyses with the additive model for 1,992,730 well-imputed SNPs using PLINK versions 1.07^45^. As the study design flow chart shows in Fig. 1, SNPs with p-value 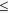
0.01 were selected as candidate SNPs in RF analysis, for which 19,701 SNPs were in the GAIN dataset and 19,524 SNPs were in the STEP dataset. Each candidate SNP was then mapped to a gene region (using NCBI build 36) if the SNP was located within the 50 kb of upstream or downstream of a gene. These SNPs were mapped to 2,832 genes in the GAIN dataset, and 3,156 genes in the STEP dataset. In total, there were 802 genes overlapped in both GWA datasets.

### Random forest procedures

To select informative risk predictors for model construction, we used the RF method for classification and building regression trees. All candidate SNPs were used to build a training model (*i.e.* 19,701 candidate SNPs in the GAIN dataset). The results from the growing of ensemble trees as a forest could provide a list of important variables for disease outcome. The RF procedures were performed using the Random Jungle package^46^, which facilitates the rapid analysis of large-scale GWA data^47^. Detailed procedures are described in the following steps:

1. Two-thirds of the subjects in the GWA dataset were taken as the training set using the bootstrap procedure, and the remaining subjects were treated as the test set.
2. Second, is the splitting step. A random subset of markers was chosen from all candidate markers without replacement. The size of each marker subset was equal to the square root of numbers of all candidate markers. The decrease in impurity for all markers was then calculated. The definition of the decrease in impurity was detailed elsewhere^46^. The marker with the best classification by the decrease in impurity was used as a node to split subjects of a training set into two distinct subsets, that is, one node split into two nodes.
3. The 2^nd^ splitting step was repeated until the tree is grown with its largest extent in the tree growing step.
4. Steps 1 to 3 were repeated to grow 5,000 classification trees to build a forest.
5. The prediction error of particular markers was estimated by permutation procedure from the test set.
6. Two indices of the RF procedures were used for the selection of relevant risk predictors, the GI and the VI. The GI represents the total decrease in impurity of the whole dataset by summing the probability of each risk predictor being chosen multiplied by the probability of a mistake in categorizing a subset. The VI means the decrease in accuracy for every predictor. To avoid the bias induced by including highly correlated candidate markers such as SNPs in LD, we used the conditional permutation scheme in the tree building procedure^48^. The conditional importance permutation groups were created, which involve all variables of a Pearson’s correlation coefficient of r 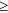
0.2, that is, the dependency structure between SNPs in linkage disequilibrium was preserved in the calculation of VI. To obtain an appropriate amount of predictors, the corresponding top ranked 200 SNPs of the GI and the conditional VI were considered as the candidate risk predictors for model construction in the next step.

### Model construction and performance evaluation

Among the candidate risk predictors identified in the GAIN dataset using the RF analysis, we applied multivariable logistic regression with variable-selection methods (i.e. stepwise selection) to select the optimal predictors and to obtain p-values, odds ratios (OR), and 95% CI. The genetic risk score for each individual was calculated by summing across all predictors in the model using the numbers of risk allele multiplied by the beta regression coefficient of each marker. The highest Youden Index^49^ was used to define the optimal cutoff point, which equals to (sensitivity + specificity)-1.

We examined the performance of the classification models by several indices. First, the discrimination capability of the established prediction model was assessed with the receiver operating characteristic (ROC) curve, and an AUROC was calculated. The ROC curve was plotted by false-positive rate versus sensitivity measure. The goodness of fit for each prediction model was assessed by the Hosmer-Lemeshow test, which calculates the difference between the predicted and the observed risk. Leave-one-out cross-validation was performed for internal validation to obtain a bias-corrected estimation of error rate in prediction. The STEP GWA dataset was used as external validation for the prediction models. Statistical analyses in this stage were performed with SAS version 9.2 (SAS Institute, Cary, NC).

### Identified significantly enriched pathways

We used the Molecular Signatures Database (MSigDB, http://www.broadinstitute.org/gsea/msigdb/annotate.jsp) to examine the common processes or the underlying biological gene sets of the selected candidate genes^50^. In the present study, we used databases including GO terms (domains in biological process, cellular component and molecular function), chromosome positional gene sets, and the curated gene sets (*e.g.* canonical pathways, *KEGG, Biocarta* and *Reactome*). In total, 3,100 collections of gene sets were available, which includes 45,956 unique genes. Enriched gene sets were identified using the hypergeometric method, with the false discovery rate less than 0.01^50^.

## Additional Information

**How to cite this article**: Chuang, L.-C. and Kuo, P.-H. Building a genetic risk model for bipolar disorder from genome-wide association data with random forest algorithm. *Sci. Rep.* **7**, 39943; doi: 10.1038/srep39943 (2017).

**Publisher's note:** Springer Nature remains neutral with regard to jurisdictional claims in published maps and institutional affiliations.

## Supplementary Material

Supplementary Table 1

## Figures and Tables

**Table 1:** The performance of discrimination ability for the union marker numbers of the top ranked in the two indices, the Gini Index and the conditional variable importance from the random forest procedure.

Top	NO. of SNPs	No. of SNPs after excluding markers in complete LD	AUROC	Hosmer- Lemeshow test
10	19	16	0.615	0.535
20	36	29	0.663	0.729
50	85	68	0.763	0.054
100	168	135	0.846	0.732
150	258	211	0.908	0.506
200	348	289	0.944	0.945

Note: **AUROC:** the area under the receiver characteristic curve; **95% C.I.:** 95% confidence interval.

*Markers in complete linkage disequilibrium (LD, D’ = 1) were removed before regression analysis and only one SNP was kept for each case of complete LD situation.

**Table 2:** Sensitivity, specificity, Youden Index, and the likelihood ratios of the risk score model in the GAIN bipolar disorder dataset as a training set.

Score	Sensitivity	Specificity	Youden Index	PPV	NPV	LR+	LR−
180	0.905	0.685	0.590	0.735	0.882	2.871	0.139
181	0.880	0.723	0.604	0.755	0.862	3.182	0.166
182	0.854	0.776	0.630	0.787	0.846	3.807	0.188
183	0.813	0.817	0.630	0.812	0.819	4.449	0.229
184	0.777	0.854	0.631	0.837	0.798	5.322	0.261
185	0.747	0.879	0.626	0.857	0.782	6.181	0.288

Note: **PPV:** positive predictive value; **NPV:** negative predictive value; **LR+**: the likelihood ratio for a positive test result; **LR−**: the likelihood ratio for a negative test result.

**Table 3:** The performance of discrimination ability for the genetic risk score model in the training set and the validation dataset.

Model construction: the GAIN dataset						Validation: the STEP dataset		
Models	AUROC	(95% C.I.)	Hosmer- Lemeshow test	Error rate*		AUROC	(95% C.I.)	Hosmer- Lemeshow test
				Controls	BPD			
289 candidate markers	0.944	(0.935–0.953)	0.933	0.208	0.220	0.702	(0.681–0.723)	0.681
121 optimal markers	0.924	(0.913–0.935)	0.458	0.193	0.209	0.639	(0.617–0.662)	0.002
The risk score variable	0.905	(0.893–0.918)	0.264	0.179	0.193	0.506	(0.482–0.530)	0.954

Note: **AUROC:** the area under the receiver characteristic curve; **95% C.I.:** 95% confidence interval.

^*^Error rate was examined by the leave-one-out cross-validation procedure.

**Table 4:** The significant gene sets for bipolar disorder based on the candidate markers from the GAIN and the STEP datasets.

Gene set name	No. of gene in gene set	No. of gene*	Dataset#	q-value
Acetyl-glucosaminyl transferase activity	16	2	STEP	3.4 × 10^−03^
Actin binding	76	3	GAIN	2.8 × 10^−03^
Actin cytoskeleton organization and biogenesis	104	3	GAIN	6.7 × 10^−03^
Actin filament	18	2	STEP	4.3 × 10^−03^
Actin filament based process	114	3	GAIN	8.6 × 10^−03^
Amine metabolic process	137	4	STEP	6.8 × 10^−03^
Anatomical structure morphogenesis	374	6	GAIN	2.6 × 10^−03^
Auxiliary transport protein activity	25	2	STEP	8.1 × 10^−03^
Axon guidance	22	2	GAIN	2.9 × 10^−03^
Axonogenesis	43	3	GAIN	5.3 × 10^−04^
Brain development	51	3	GAIN	8.8 × 10^−04^
Calcium channel activity	33	2	GAIN	6.5 × 10^−03^
Calcium ion transport	27	2	GAIN	4.4 × 10^−03^
Calmodulin binding	25	2	STEP	8.1 × 10^−03^
Carbohydrate binding	72	3	STEP	7.2 × 10^−03^
Cation transport	146	4	STEP	8.4 × 10^−03^
Cell migration	93	3	GAIN	4.9 × 10^−03^
Cell surface	76	3	STEP	8.3 × 10^−03^
Cellular morphogenesis during differentiation	49	3	GAIN	7.8 × 10^−04^
Channel regulator activity	23	2	STEP	6.9 × 10^−03^
Chr12q23	78	3	STEP	8.9 × 10^−03^
Chr2p23	75	4	STEP	7.6 × 10^−04^
Chr2q23	25	2	STEP	8.1 × 10^−03^
Chr3p14	56	3	STEP	3.6 × 10^−03^
Chr4q34	19	2	STEP	4.7 × 10^−03^
Chr6q13	23	2	STEP	6.9 × 10^−03^
Chr6q26	16	2	STEP	3.4 × 10^−03^
Cytoplasmic membrane bound vesicle	112	3	GAIN	8.2 × 10^−03^
Cytoplasmic vesicle	116	3	GAIN	9.0 × 10^−03^
Cytoskeletal protein binding	158	5	GAIN	3.0 × 10^−04^
Cytoskeleton	361	6	GAIN	2.2 × 10^−03^
Di-, tri-valent inorganic cation transport	32	2	GAIN	6.2 × 10^−03^
Endocytic vesicle	14	2	GAIN	1.2 × 10^−03^
Enzyme regulator activity	314	6	STEP	7.6 × 10^−03^
Establishment of localization	852	12	STEP	2.5 × 10^−03^
G protein signaling coupled to IP3 second messenger phospholipase C activating	41	2	GAIN	1.0 × 10^−02^
GABA receptor activity	11	2	STEP	1.6 × 10^−03^
Generation of neurons	83	3	GAIN	3.6 × 10^−03^
Integrin binding	30	2	GAIN	5.4 × 10^−03^
Ion transport	184	5	STEP	3.4 × 10^−03^
KEGG-Arrhythmogenic right ventricular cardiomyopathy	76	5	GAIN	9.0 × 10^−06^
KEGG-Calcium signaling pathway	178	4	GAIN	4.2 × 10^−03^
KEGG-Cardiac muscle contraction	80	4	GAIN	2.2 × 10^−04^
KEGG-Dilated cardiomyopathy	92	4	GAIN	3.7 × 10^−04^
KEGG-Hypertrophic cardiomyopathy HCM	85	4	GAIN	2.7 × 10^−04^
Membrane	1942	20	GAIN	2.1 × 10^−05^
Membrane bound vesicle	114	3	GAIN	8.6 × 10^−03^
Membrane organization and biogenesis	133	4	STEP	6.1 × 10^−03^
Membrane part	1633	13	GAIN	6.6 × 10^−03^
Neurite development	53	3	GAIN	9.8 × 10^−04^
Neurogenesis	93	3	GAIN	4.9 × 10^−03^
Neuron development	61	3	GAIN	1.5 × 10^−03^
Neuron differentiation	76	3	GAIN	2.8 × 10^−03^
Neuropeptide binding	23	2	GAIN	3.2 × 10^−03^
Neuropeptide receptor activity	22	2	GAIN	2.9 × 10^−03^
Nitrogen compound metabolic process	150	4	STEP	9.3 × 10^−03^
Plasma membrane	1393	14	GAIN	5.4 × 10^−04^
Plasma membrane part	1141	10	GAIN	9.0 × 10^−03^
RAS guanyl nucleotide exchange factor activity	18	2	STEP	4.3 × 10^−03^
Reactome-Depolarization of the presynaptic terminal triggers the opening of calcium channels	12	2	GAIN	8.6 × 10^−04^
Reactome-Neurotransmitter release cycle	28	2	GAIN	4.7 × 10^−03^
Reactome-Transmission across chemical synapses	130	4	GAIN	1.4 × 10^−03^
Receptor mediated endocytosis	33	2	GAIN	6.5 × 10^−03^
Regulation of action potential	17	2	STEP	3.8 × 10^−03^
Response to external stimulus	306	6	STEP	6.7 × 10^−03^
ST interleukin 4 pathway	26	2	STEP	8.8 × 10^−03^
System process	558	8	GAIN	1.0 × 10^−03^
Transmembrane receptor activity	411	8	STEP	1.9 × 10^−03^
Transport	778	11	STEP	3.6 × 10^−03^
Voltage-gated calcium channel activity	18	2	GAIN	2.0 × 10^−03^
Voltage-gated calcium channel complex	15	2	GAIN	1.4 × 10^−03^

**q-value:** the value of false discovery rate.

^*^Number of gene in overlap.

^#^The dataset of significant gene set.
