# Supplementary material for: Building a genetic risk model for bipolar disorder from genome-wide association data with random forest algorithm
Source: Sci Rep. 2017 Jan 3;7:39943. doi: 10.1038/srep39943 (PMC5206749; doi:10.1038/srep39943)
Supplement: Supplementary Table 1 [file srep39943-s1.doc]

**Building a genetic risk model for bipolar disorder from genome-wide association data with random forest algorithm**

Li-Chung Chuang, Po-Hsiu Kuo

**Supplementary Table 1. The performance of discrimination for the genetic risk score model in the STEP dataset and the validation dataset in the GAIN dataset**

| **Model construction** | | | | | | |  | **Validation** | | | |
| --- | --- | --- | --- | --- | --- | --- | --- | --- | --- | --- | --- |
| **Training** |  | **AUROC** | **(95% C.I.)** | **Hosmer-**  **Lemeshow test** | **Error rate** | |  | **Test** | **AUROC** | **(95% C.I.)** | **Hosmer-**  **Lemeshow test** |
| **Dataset** | **Model** |  |  |  | **Controls** | **BPD** |  | **Dataset** |  |  |  |
| STEP | 312 SNPs | 0.934 | (0.925-0.944) | 0.3196 | 0.209 | 0.235 |  | GAIN | 0.732 | (0.711-0.754) | 0.8006 |
| STEP | 142 SNPs | 0.911 | (0.900-0.923) | 0.0557 | 0.215 | 0.208 |  | GAIN | 0.660 | (0.636-0.683) | 0.6375 |

**Note: AUROC:** the area under receiver characteristic curve; **95 % C.I.:** 95 % confidence interval
